# A natriuretic peptide from *Arabidopsis thaliana* (AtPNP-A) can modulate catalase 2 activity

**DOI:** 10.1038/s41598-020-76676-0

**Published:** 2020-11-12

**Authors:** Ilona Turek, Janet Wheeler, Sebastian Bartels, Jolanta Szczurek, Yu Hua Wang, Phil Taylor, Chris Gehring, Helen Irving

**Affiliations:** 1 Biomolecular Laboratory, Division of Biological and Environmental Sciences and Engineering, King Abdullah University of Science and Technology, 4700, Thuwal, 2395-6900 Saudi Arabia; 2 Department of Pharmacy and Biomedical Sciences, La Trobe Institute for Molecular Science, La Trobe University, Bendigo, VIC 3552 Australia; 3 Monash Institute of Pharmaceutical Sciences, Monash University, Melbourne, VIC 3052 Australia; 4 AgriBio, La Trobe University, Bundoora, VIC 3083 Australia; 5 Zurich-Basel Plant Science Center, Department of Environmental Sciences - Botany, University of Basel, 4056 Basel, Switzerland; 6 Department of Endocrinology, Metabolism and Internal Medicine, Poznan University of Medical Sciences, 60-512 Poznan, Poland; 7 Department of Chemistry, Biology and Biotechnology, University of Perugia, 06121 Perugia, Italy

**Keywords:** Biochemistry, Plant sciences

## Abstract

Analogues of vertebrate natriuretic peptides (NPs) present in plants, termed plant natriuretic peptides (PNPs), comprise a novel class of hormones that systemically affect salt and water balance and responses to plant pathogens. Several lines of evidence indicate that *Arabidopsis thaliana* PNP (AtPNP-A) affects cellular redox homeostasis, which is also typical for the signaling of its vertebrate analogues, but the molecular mechanism(s) of this effect remains elusive. Here we report identification of catalase 2 (CAT2), an antioxidant enzyme, as an interactor of AtPNP-A. The full-length AtPNP-A recombinant protein and the biologically active fragment of AtPNP-A bind specifically to CAT2 in surface plasmon resonance (SPR) analyses, while a biologically inactive scrambled peptide does not. In vivo bimolecular fluorescence complementation (BiFC) showed that CAT2 interacts with AtPNP-A in chloroplasts. Furthermore, CAT2 activity is lower in homozygous *atpnp-a* knockdown compared with wild type plants, and *atpnp-a* knockdown plants phenocopy *CAT2*-deficient plants in their sensitivity to elevated H_2_O_2_, which is consistent with a direct modulatory effect of the PNP on the activity of CAT2 and hence H_2_O_2_ homeostasis. Our work underlines the critical role of AtPNP-A in modulating the activity of CAT2 and highlights a mechanism of fine-tuning plant responses to adverse conditions by PNPs.

## Introduction

In plants abiotic and biotic stress triggers highly complex stimulus-specific cellular and systemic signals and responses consisting of many different components including receptors, sensors, plant hormones, notably abscisic acid (ABA), jasmonic acid and (second) messengers such as calcium and cyclic mononucleotides^1–4^. Perception of many phytohormones by different types of intracellular and extracellular receptors is well documented. For instance, ABA is not only perceived by a family of nucleocytoplasmic PYR/PYL/RCAR (PYLs) that bear unequivocal hallmarks of the *bona fide* ABA receptors^5,6^, but also by plasma membrane guard cell outward rectifying potassium channel (GORK)^7^, and possibly other receptors of ABA are still to be discovered.

Similarly, identification of novel receptors targeted by an increasing number of peptidic plant hormones discovered in the last few decades^8,9^ is anticipated. One of those, the plant natriuretic peptides (PNPs), are a group of systemically mobile signals^10^ that have a role in the maintenance of salt and water balance^11,12^. An *A. thaliana* PNP, termed AtPNP-A (At2g18660; Q9ZV52), has been implicated in several physiological processes ranging from the regulation of stomatal aperture^13^, osmoticum-dependent volume changes^13,14^ and modulation of developmental stage- and tissue-specific ion fluxes^15^, to immune responses^16,17^. Much like vertebrate natriuretic peptides (NPs)^18^, many of the effects elicited by AtPNP-A involve rapid elevation of 3′,5′-cyclic guanosine monophosphate (cGMP)^13,19^, and increasing evidence suggests that reactive oxygen species (ROS) are also secondary messengers in the transduction of AtPNP-A signals^20^. Nevertheless, understanding of the molecular mechanisms by which PNPs exert their functions is limited by lack of comprehensive studies reporting sets of proteins that interact with PNPs to modulate levels of secondary messengers, including cGMP or ROS, which are relevant elements of plant defense responses regulated by PNPs. Although at least two receptors perceiving AtPNP-A have been identified^16,21^, and at least one of them catalyzes generation of cGMP upon binding of AtPNP-A^21^, determination of the signaling underlying AtPNP-A-dependent modulation of ROS has not been attempted. In particular, abiotic and biotic stress can cause rapid increases in hydrogen peroxide (H_2_O_2_) levels which in turn activate physiological responses, including seed germination^22,23^, stomatal aperture movement regulation^24–27^, programmed cell death^28,29^ and others^30^. Many of these events are known to be modulated by PNPs^10,13,16,17,20,31,32^. Since these processes have implications on salt stress acclimation^33, 34^, drought tolerance^35,36^, and plant responses to other abiotic^37^ and biotic^38^ stresses, understanding how PNPs affect ROS could enhance elucidation of plant stress responses in agricultural settings.

In this study, we set out to identify interactors of AtPNP-A to elucidate possible direct links between the hormone and changes in cellular redox homeostasis and identified Catalase 2 (CAT2; At4g35090; P25819) as a direct binding partner of AtPNP-A. CAT2 has been reported as the major enzyme involved in detoxifying ROS in the photosynthetic tissues^39^ essential for optimal development of C_3_ plants grown in air^40^. Using a series of biochemical and physiological approaches we investigated the specificity of this binary interaction and its biological relevance. Synthetic peptide containing amino acids corresponding to the evolutionarily conserved active site of AtPNP-A binds specifically not only to *A. thaliana* CAT2, but also to animal-derived catalase, underlying general significance of the interaction between NPs and catalases in different kingdoms. We show that AtPNP-A modulates enzymatic activity of CAT2, the interaction occurs in chloroplasts, and *atpnp-a* knockdown mutant displays differential responses to redox stress, phenocopying *cat2* knockout mutant plants. The interaction between PNPs and CAT2 affects cellular H_2_O_2_, thereby possibly modulating biotic and abiotic stress responses.

## Methods

### Plant materials and growth conditions

Seeds of *Arabidopsis thaliana* (Col-0) and mutants carrying T-DNA insertion in either *CAT2* (*cat2-2*; SALK_057998) or *AtPNP-A* (*atpnp-a*; SALK_000951)^41^, from the European Arabidopsis Stock Centre (uNASC; https://arabidopsis.info), were surface-sterilized and vernalized, sown in Jiffy peat pellets and grown at 23 °C in 16 h of light (200 μmol s^−1^ m^−2^) per day or on Murashige-Skoog agar plates and grown at 23 °C in 16 h of light (100 μmol s^−1^ m^−2^) per day for 10 days. Plant genotyping is detailed in Supplementary Table S1. Determination of the site of the T-DNA insertion in the *atpnp-a* homozygous mutant line was done by sequencing of the purified PCR fragment containing the junction of the T-DNA and plant genomic DNA obtained with the use of the mutant allele-specific primers given in Supplementary Table S1. *Nicotiana tabacum* seeds were germinated on soil (Debco seed raising mix:vermiculite, 3:1). At three weeks seedlings were transferred to three plants per pot (Debco soil mix:vermiculite, 3:1). Plants were grown under long day conditions (16 h light, 8 h dark) at 22 °C.

### Amino acid sequence alignment

Amino acids sequences of *A. thaliana* AtPNP-A (NP_849979.1) orthologs expressed in *S. tuberosum* (StPNP-A; XP_015162511.1) and in *N. tabaccum* (NtPNP-A; XP_016448760) were derived from NCBI with pBLAST search using AtPNP-A sequence without the signal peptide (119–130) as a query. The alignment was performed using ClustalW software.

### Transmission electron microscopy (TEM)

Samples for transmission electron microscopy (TEM) were prepared according to^42^. Briefly, 2 mm sections of potato were plunged into liquid propane pre-cooled on liquid nitrogen at approximately − 200 °C. Samples were freeze substituted for 3 weeks at − 70 °C in 1% (w/v) glutaraldehyde in 2,2-dimethoxypropane, acetone and methanol. Upon bringing to room temperature, the samples were rinsed in solvent and infiltrated with LR Gold resin over a further 2 weeks. Samples were then polymerized at room temperature with a catalyst, and ultrathin sections were cut on an ultramicrotome using a diamond knife. Sections (80 nm thick) were picked up on pioloform coated gold grids and dried. These grids were immune-stained, upon BSA/PBS blocking, with the polyclonal anti-human ANP antibody^43^, followed by goat anti-rabbit antibody pre-conjugated to 40 nm gold nanoparticles. Grids were examined in a transmission electron microscope (Phillips). Photos were captured on negatives and processed in the dark room, printed, and scanned to digital. If more contrast was then needed to examine cell details, the grids were post-stained in uranyl acetate and lead citrate. Controls for immunolabeling included omission of antibody and usage of an irrelevant antibody (monoclonal antibody directed against grass allergens).

### Verification of biological activity of synthetic peptides

**Figure 1:**
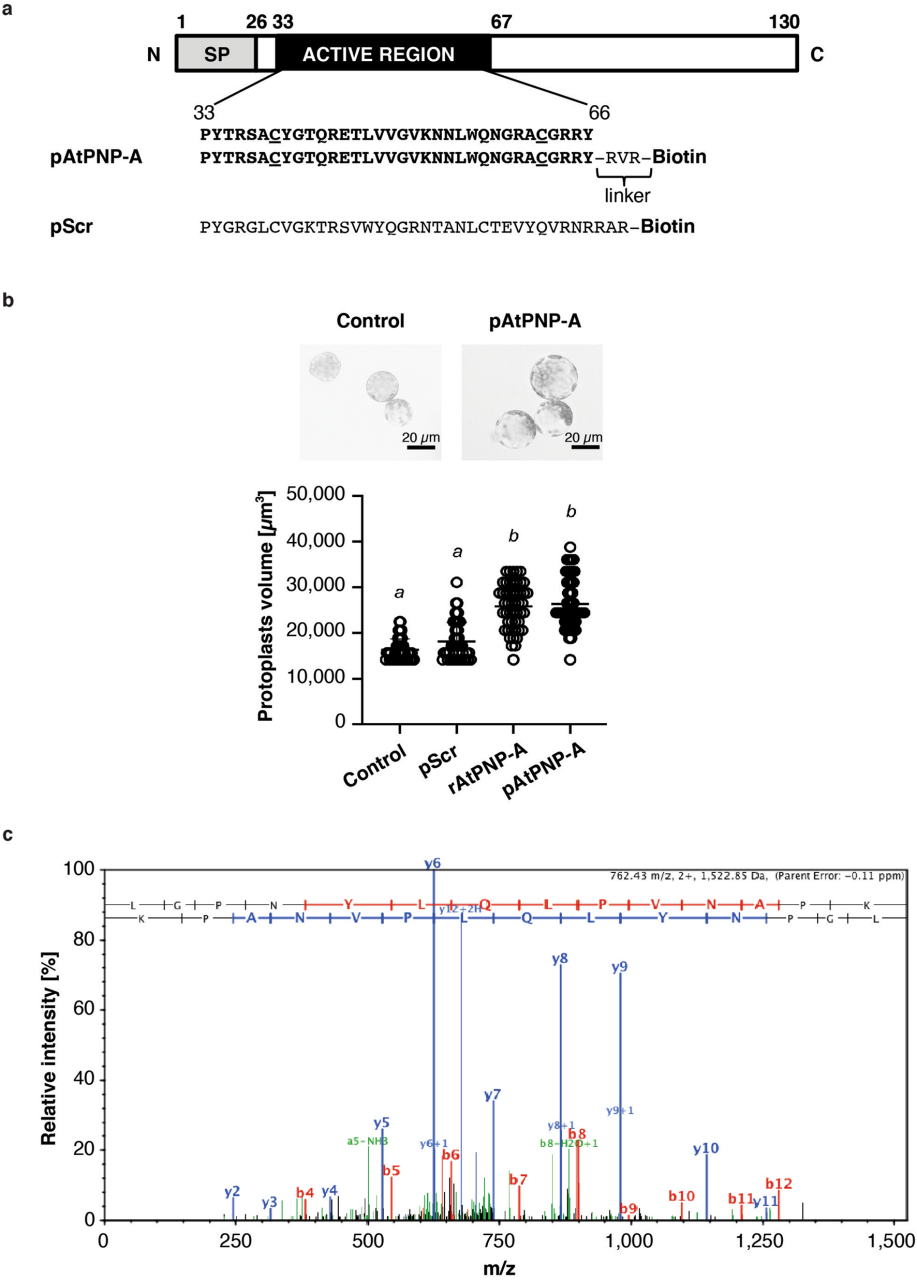
Biologically active peptide containing the active region of AtPNP-A protein binds to CAT2. (**a**) Domain organization of AtPNP-A and the amino acid sequences of C-terminally biotinylated peptides used in affinity chromatography-based experiments—a peptide containing the active site of AtPNP-A (indicated as pAtPNP-A) or the corresponding scrambled peptide (indicated as pScr). Cysteine residues forming a disulfide bond, characteristic to natriuretic peptide (NP)-like molecules, are underlined. SP, signal peptide. (**b**) Assaying biological activity of the AtPNP-A peptide (pAtPNP-A) and purified recombinant protein (rAtPNP-A). *A. thaliana* (Col-0) mesophyll cell protoplasts suspended in 0.4 M mannitol were treated with either water or 100 nM pScr (negative controls) or with 100 nM pAtPNP-A or with 1 μg mL^−1^ of rAtPNP-A protein for 20 min at room temperature. In each treatment, 50 randomly selected protoplasts with diameter > 20 µm were included in quantitative analysis (scale bar = 20 µm). Protoplast volume was measured and the data obtained from an exemplar experiment are plotted. Columns with different superscript (*a* and *b*) indicate significantly different results (mean ± SD, one way ANOVA followed by Tukey–Kramer multiple comparison test, *n* = 50, *P* < 0.0001). (**c**) Exemplar MS/MS spectrum of a unique tryptic peptide of CAT2 (At4g35090) protein.

Peptides containing amino acid sequence of the active region of AtPNP-A and corresponding scrambled peptides, having the same amino acid composition but in a randomized order (Fig. 1a), with or without C-terminal biotin tag, were synthesized by GenScript (Piscataway, USA) at the purity level > 95% verified with HPLC and their biological activity was verified as described previously^21^.

### Identification of AtPNP-A interactors by protein-peptide cross-linking followed by affinity-based isolation and LC–MS/MS analysis

Cross-linking experiments followed by affinity-based isolation and LC–MS/MS identification of AtPNP-A interactors were performed on *A. thaliana* (Col-0) wild type (WT) mesophyll cell protoplasts (MCPs) as described in^44^.

### Identification of AtPNP-A interactants by yeast two-hybrid (Y2H) screen

The Y2H screen using AtPNP-A as bait was carried out by Dualsystems Biotech AG (Zurich, Switzerland). The bait construct for the screening was made by subcloning the cDNA fragment encoding exported portion of AtPNP-A (amino acids 26 to 130) (Fig. 1a) into the pLexA-DIR vector (Dualsystems Biotech AG). The bait construct was transformed into the strain NMY32 (MATa his3Δ200 trp1-901 leu2-3,112 (lexAop)_8_-ADE2 LYS2::(lexAop)_4_-HIS3 URA3::(lexAop)_8_-lacZ GAL4) using standard procedures^45^. Correct expression of the bait was verified by western blotting of cell extracts using a mouse monoclonal antibody directed against the LexA domain (Dualsystems Biotech AG). The absence of self-activation was verified by co-transformation of the bait together with a control prey and selection on minimal medium lacking tryptophan, leucine, and histidine (selective medium). For the Y2H screen, the bait was co-transformed together with a normalized *A. thaliana* universal P02403 cDNA library (Dualsystems Biotech AG) into NMY32. 3.1 × 10^6^ transformants were screened, yielding 96 transformants that grew on selective medium. Positive transformants were tested for β-galactosidase assay and only those that showed β-galactosidase activity were considered true positives. Library plasmids were isolated from positive clones. The identity of positive interactants was determined by sequencing. DNA sequences translated in all three reading frames and searching against the SwissProt database using the BLASTX algorithm (https://blast.ncbi.nlm.nih.gov).

### Association of AtPNP-A with chloroplasts

Protoplasts were isolated from Arabidopsis leaves, transiently transfected with the full-length AtPNP-A (GFP:signal peptide AtPNP-A fusion expression vector)^46^ and imaged with a LSM Pascal confocal microscope (Zeiss). Green channel (FITC) and red channel (TR) were selected together (multi tracks) for scanning. Images were stored and viewed in the LSM Image Browser software (Zeiss). A crude chloroplast preparation was obtained from WT Arabidopsis leaves according to^47^. Proteins from chloroplasts and WT untransfected protoplasts were extracted as previously described in^46^ and protein concentrations were measured using the Quant-iT protein assay kit (Invitrogen). Western analysis was according to a standard protocol^48^. The primary anti-AtPNP antibody was prepared against peptides representing amino acids 44–55 of AtPNP-A as described in^49^. Immunoreactive bands were visualized by incubating with TM/B peroxidase substrate solution (Chemicon/Millipore) for about 5 min.

### Oxidative burst measurement

Leaf discs (0.196 cm^2^) of 4-week-old WT plants were incubated overnight floating on 0.1 mL water in 96-well titer plate, with one disc per well. The following day the leaf discs were pre-treated with 100 nM rAtPNP-A or mock for 30 min. For ROS detection horseradish peroxidase and luminol (Sigma-Aldrich) were added to a final concentration of 10 μg mL^−1^ and 100 μM, respectively. Luminescence was measured directly after addition of either 1 μM of flg22 or 100 nM rAtPNP-A in a MicroLumat LB96P plate reader (Berthold Technologies) for 1 h and is shown in relative light units.

### Bimolecular fluorescence complementation (BiFC) of AtPNP-A and CAT2

The full-length *A. thaliana* AtPNP-A (At2g18660) coding region entry plasmid pENTRY*AtPNP-A*^46^ and pCR8/GW-TOPO*CAT2* were recombined separately with both pSITE-nEYFP-C1 [GenBank Accession Number GU734651] and pSITE-cEYFP-C1 [GenBank Accession Number GU734652] to create pNeYFP-*AtPNP-A*, pCeYFP-*AtPNP-A*, pNeYFP-*CAT2* and pCeYFP-*CAT2*, respectively. Each plasmid has either the N-terminal (NeYFP) or C-terminal (CeYFP) fragment of eYFP followed by either *AtPNP-A* or *CAT2* coding region driven by the 35S plant expression promoter. After sequence was confirmed each plasmid was transformed separately into *Agrobacterium tumefaciens* strain GV3101 (pMP90). Tobacco leaves were infiltrated as previously described in^50^. Briefly*, A. tumefaciens* strains grown in 2YT medium were harvested by centrifugation and resuspended in infiltration medium (10 mM MES pH 5.6, 10 mM MgCl_2_, 150 µM acetosyringone) to a final OD_600_ of 0.2. Equal volumes of infiltration media containing relevant BiFC strains were mixed and infiltrated into the underside of tobacco leaves using a needleless syringe. Leaves were harvested after 3 days to isolate protoplasts following^51^ and then immediately imaged.

BiFC images were captured at the La Trobe University LIMS BioImaging Facility using a LSM 510 confocal laser scanning microscope (Zeiss), with a × 40/1.2 water immersion objective. The natural red autofluorescence of chlorophyll was used as a marker for chloroplasts^52^. Excitation/emission wavelengths were 488/505–530 nm for YFP and 561/650 nm long pass for red fluorescence. At least three images containing one or more protoplasts were taken for each of the plasmid construct combinations. Each image captured three separate filter scans, including YFP, red fluorescence and bright field. Post-processing of images was performed with ImageJ version 1.48 software (https://imagej.nih.gov/ij/). For each image the contrast for each panel was set at automatic contrast. A merged image was generated for reference with a 10 µm scale bar. The red fluorescence only panel was used to encircle ten chloroplasts as region of interest (ROI), then the YFP fluorescence (BiFC) calculated for each ROI was divided by the red fluorescence calculated for the same ROI. All BiFC/red fluorescence points were plotted using GraphPadPrism7 (GraphPadSoftware). Mean and standard error were calculated for each BiFC combination. All statistical analyses were performed using GraphPadPrism7.

### Prediction of protein associations and protein–protein docking

The structures of AtPNP-A as well as CAT2 monomer were predicted using the iterative threading assembly refinement (I-TASSER; https://zhanglab.ccmb.med.umich.edu/I-TASSER/) method^53^. Protein–protein docking was performed using ClusPro (version 2.0; https://cluspro.bu.edu/publications.php)54. The models were analyzed and visualized using UCSF Chimera (version 1.10.2)^55^.

### Preparation of recombinant proteins

N-terminally 6xHis-tagged AtPNP-A and CAT2 proteins were expressed in BL21 (DE3) One Shot *E. coli* cells (Life Technologies), purified by affinity chromatography with Ni–NTA beads (Qiagen) and HisTrap HP column (GE Healthcare Lifesciences) as described in^21^. The purity of preparations was verified on 12.5% SDS-PAGE, stained with Coomassie Brilliant Blue (Bio-Rad). Protein identities were confirmed in MS analysis, and protein concentration was determined according to the method of Bradford using BSA as a standard.

### Surface plasmon resonance (SPR) analyses

SPR experiments were performed at 20 °C on Biacore T100 instrument operated using Biacore T100 control software (version 2.0.2, GE Healthcare Lifesciences) using Series S CM5 or NTA sensor chips as described in^21^. Kinetic analyses were performed at the flow of 100 μL min^−1^ with pAtPNP-A (at 3.78 μM and consecutive twofold dilutions; 11 injections included) employed as a ligand and bovine liver CAT (C-40; Sigma) used as an analyte and immobilized on the active surface of the Series S CM5 sensor chip with amine coupling kit, while the surface regeneration was done with solution of glycine, pH 2.0, and the final sensorgram was generated with Scrubber (BioLogic Software Pty Ltd).

### Isothermal titration calorimetry (ITC)

ITC experiments were undertaken using MicroCal iTC200 (Malvern Panalytical) calorimeter. The titrations were conducted at 25 °C in PBS buffer at pH 7.4, with 100 μM pAtPNP-A used as a titrant. Total number of injections was set at 30, reference power was 11 μcal s^−1^, initial delay was 60 s, and stirring speed was 750 rpm.

### Detection of CAT activities and isozymes analysis

Enzymatic activity of CAT isozymes and total CAT activities of protein extracted from WT and *atpnp-a* or *cat2-2* mutant lines, when indicated sprayed with 1 nM pAtPNP-A or pScr 20 min before harvesting, were determined zymographically and spectrophotometrically, as described in^56^. Relative quantification of intensity of bands corresponding to different CAT isoforms was performed with ImageJ version 1.48 (https://imagej.nih.gov/ij/). Enzymatic activity of the rCAT2 in the presence of absence of 100 nM rAtPNP-A, pAtPNP-A or pScr were determined with the use of Amplex Red catalase assay kit (Invitrogen) according to the manufacturer’s instructions.

### Germination assay

Screening for H_2_O_2_ tolerance of *cat2-2*, *atpnp-a*, and WT, the seeds were germinated on Murashige-Skoog agar plates supplemented with 1% (w/v) sucrose and 3 mM H_2_O_2_. The plates were incubated in growth chambers under conditions described above and photographed on day 10. Each assay was performed at least three times in triplicate, with 50 seeds sown per each line, and the results of three independent experiments per treatment (mean ± SD) were plotted.

## Results

### Identification of CAT2 as a candidate interactor of AtPNP-A

We sought biologically relevant binding partners of AtPNP-A using two complementary approaches to identify in vivo protein–protein interactions, namely protein-peptide cross-linking followed by affinity-based isolation and liquid chromatography-tandem mass spectrometry (LC–MS/MS) analysis as well as yeast two-hybrid (Y2H) screen. Since common organellar localization of interacting proteins is a pre-requisite for the binding event to occur, we first verified subcellular localization of PNPs (Supplementary Fig. S1b–e). Initially, we examined subcellular localization of PNP, termed StPNP, natively expressed in potato (*Solanum tuberosum*), due to the large cell size and responsiveness of this system to both atrial natriuretic peptide (ANP) and immunoreactive plant PNPs^19^, as the active region of StPNP is highly conserved (Supplementary Fig. S1a). Electron microscopy suggested that PNP localizes not only to the cell wall, but can also be found in starch bodies in potato plastids (Supplementary Fig. S1b,c). Furthermore, confocal microscopy of *A. thaliana* transiently transfected with green fluorescent protein (GFP)-fused AtPNP-A confirmed co-localization of PNP in chloroplasts (Supplementary Fig. S1d), consistent with localization of native AtPNP-A both in chloroplasts and mesophyll cell protoplasts (MCPs) isolated from wild type (WT) *A. thaliana* (Supplementary Fig. S1e). For that reason, cross-linking experiments were performed on MCPs and making use of a C-terminally biotinylated synthetic peptide of AtPNP-A (pAtPNP-A) containing the amino acid sequence of its experimentally determined active region (amino acids 33–66)^13,43^ (Fig. 1a–b). The pAtPNP-A dependent protoplast swelling response is significantly greater than the response elicited by the scrambled peptide (pScr) (Fig. 1b) and comparable to that elicited by an equimolar amount of the recombinant AtPNP-A (rAtPNP-A) (Fig. 1b). The biologically active pAtPNP-A was subsequently used in binding experiments performed in *A. thaliana* (Col-0) MCPs in the presence of cross-linking agents enabling identification of weak interactors of AtPNP-A. Cross-linking experiments with biotin in the presence of the cross-linking agent excluded nonspecific interactors, whereas binding of proteins to the biotinylated pAtPNP-A in the absence of the cross-linker allowed identification of interactions lost due to the presence of the cross-linker. Subsequent affinity-based isolation of pAtPNP-A (or biotin) with bound interactors followed by LC–MS/MS identification enabled relative quantification of spectral counts corresponding to peptides matching putative binding partners of pAtPNP-A^44^.

To further increase confidence in the candidate preys being true positives, a Y2H screen was performed with AtPNP-A (amino acids 26–130), which includes the active region of AtPNP-A corresponding to the amino acid sequence of pAtPNP-A, as a bait (Fig. 1a). The Y2H assay identified 25 candidate AtPNP-A preys, 13 of which showed weak interaction strength (Table 1). Only two proteins were identified as putative interactors of AtPNP-A with both methods: catalase 2 (CAT2; At4g35090; P25819) (Fig. 1c) and rubisco activase (RCA; At2g39730; Q0WLM1) (Table 1). CAT2 was identified by LC–MS/MS only in samples containing pAtPNP-A, while the RCA was also abundant in control samples without the peptide^44^. PNPs are known to modulate redox signaling^20^, therefore we tested if flg22, a 22-amino acid peptide derived from bacterial flagellin recognized by the FLS2 receptor^57^ induced changes in redox signaling. AtPNP enhances responses to flg22 (Supplementary Fig. S2), suggestive of a redox effect so we undertook to test the binding between AtPNP-A and the antioxidant enzyme CAT2.

**Table 1:** Proteins identified as candidate direct interactors of AtPNP-A in the yeast two-hybrid (Y2H) analysis.

Protein	AGI	Strength of binding
ERF7, ethylene response factor 7	At3g20310.1	Strong
ERP/AP2, ethylene response factor	At5g07580.1	Strong
RAP2.12, ethylene response factor	At1g53910.1	Strong
IGPD, imidazole glycerol-phosphate dehydratase	At3g22425.1	Strong
SHA1, shoot apical meristem arrest 1	At5g63780.1	Strong
PLL4, poltergeist like 4	At2g28890.1	Strong
RCA, rubisco activase	At2g39730.1	Strong
MAC5S, MOS4-associated complex subunit 5C	At5g07060.1	Strong
HISN5B, histidine biosynthesis 5B	At4g14910.1	Moderate
NUDX1, nudix hydrolase 1	At1g68760.1	Moderate
Protein containing PAM2 motif	At4g14270.1	Moderate
NAD(P)-binding Rossmann-fold superfamily protein	At1g07440.1	Moderate
ER, enhanced of rudimentary homologue, ATER	At5g10810.1	Weak
PGR1, photosynthetic electron transfer C	At4g03280.1	Weak
ADH5, alcohol dehydrogenase 2	At5g43940.1	Weak
CAT2, catalase 2	At4g35090.1	Weak
CRF6, cytokinin response factor 6	At3g61630.1	Weak
Expr, expansin-like B1	At4g17030.1	Weak
FED A, ferredoxin 2	At1g60950.1	Weak
CLA, 1-deoxy-d-xylulose-5-phosphate synthase	At4g15560.1	Weak
Calcium-binding EF-hand family protein	At2g41410.1	Weak
Basic-leucine zipper (bZIP) TF family protein	At2g31370.1	Weak
SPL8, squamosal promoter binding protein-like 8	At1g02065.1	Weak
Unknown protein	At5g39570.1	Weak
COL1, constans-like 1	At5g15850.1	Weak

### AtPNP-A specifically binds CAT2 in vitro

**Figure 2:**
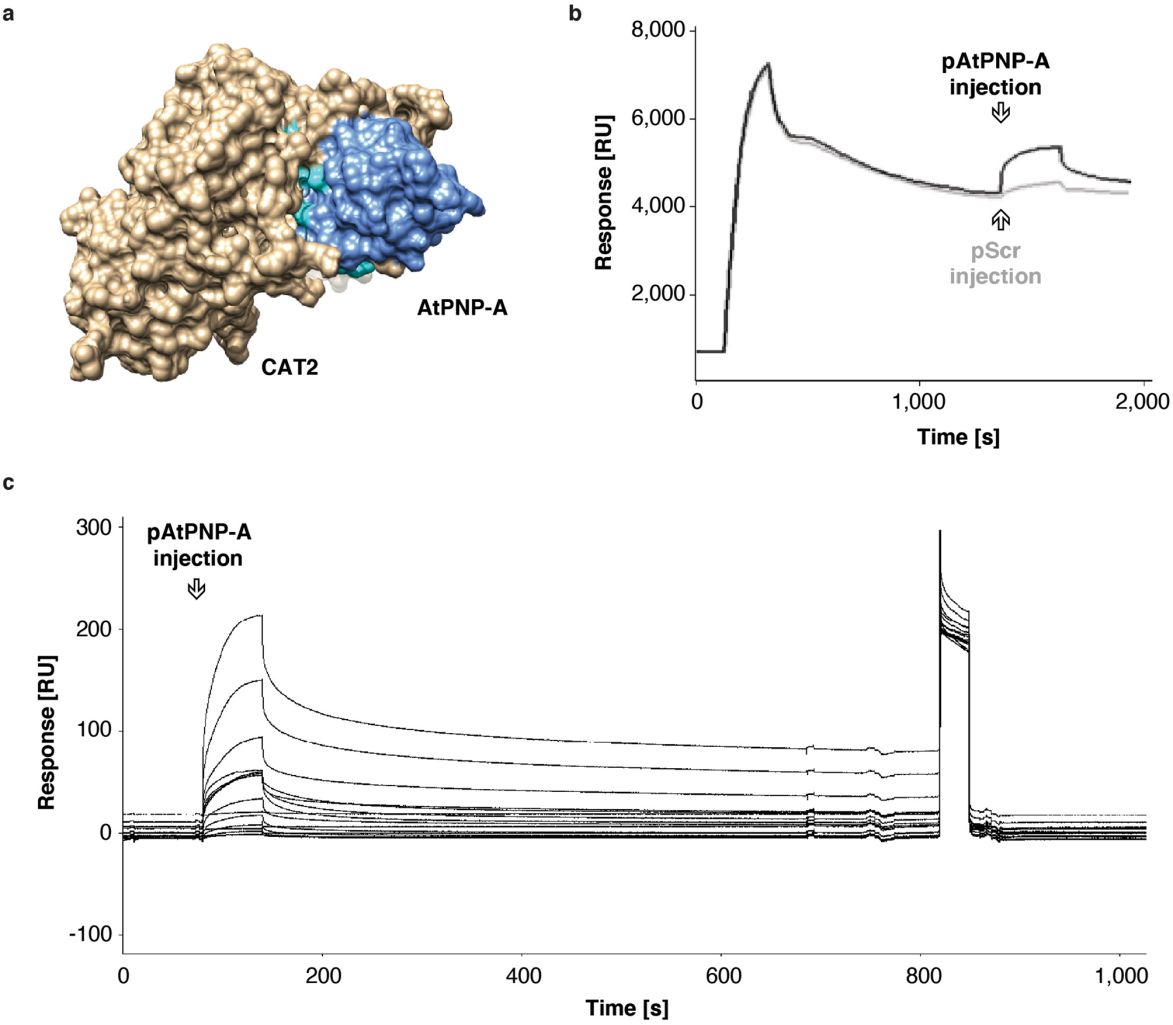
AtPNP-A directly interacts with Arabidopsis CAT2 and bovine liver CAT in vitro. (**a**) Molecular docking of AtPNP-A and CAT2. Surface model depicts predicted docking of AtPNP-A (blue), with its active region (cyan), and CAT2 monomer (tan). The structures of AtPNP-A as well as CAT2 monomer were predicted using the iterative threading assembly refinement (I-TASSER; version 5.1; https://zhanglab.ccmb.med.umich.edu/I-TASSER/) method^53^. Protein–protein docking was performed using ClusPro (version 2.0; https://cluspro.bu.edu/publications.php)^54^. The models were analyzed and visualized using UCSF Chimera (version 1.10.2; https://www.cgl.ucsf.edu/chimera/)^55^. (**b**) Exemplar sensorgrams depicting referenced binding response of pAtPNP-A or pScr with CAT2 recombinant protein immobilized on the active surface of the NTA sensor chip. Reference surface of the NTA chip was not modified, according to the manufacturer’s instructions, and did not carry the recombinant protein. In both analyses the ligand was immobilized at the same level (app. 4,500 RU), analytes are injected at the same concentration and conditions of runs kept constant. (**c**) Exemplar sensorgrams depicting referenced binding response in kinetic analysis of binding between pAtPNP-A (3.78 μM and consecutive two-fold dilutions, as in Supplementary Table S2) and bovine liver CAT immobilized on the active surface of the CM5 sensor chip. Reference surface of the NTA chip was not modified, according to the manufacturer’s instructions, and did not carry any protein.

Protein docking simulations making use of the 3D structure models of AtPNP-A and CAT2 predict interaction of AtPNP-A with the C-terminal portion of CAT2 monomer (Fig. 2a). To assess specificity of any binding of AtPNP-A to CAT2, the proteins were expressed as recombinants, purified (Supplementary Fig. S3a), and in vitro binding of the recombinant proteins measured in both ligand—analyte configurations with surface plasmon resonance (SPR) (Supplementary Fig. S3b,c). The specificity of the interaction between the active region of AtPNP-A and purified CAT2 recombinant (rCAT2) is confirmed in analyses making use of the NTA sensor chip, where the His-tagged rCAT2 is immobilized, while the biologically active pAtPNP-A or the corresponding biologically inactive pScr peptide injected through the flow cells, resulting in a significant accumulation of the biologically active analyte (Fig. 2b) and only negligible binding of pScr (Fig. 2b). This excludes the possibility of a nonspecific interaction between pAtPNP-A and CAT2 that would occur solely on a basis of a nonspecific charge effect residing in the primary structure of the peptide. Binding analyses between pAtPNP-A and bovine CAT and pAtPNP-A performed in both ligand—analyte configurations using the CM5 sensor chip with amine-coupling chemistry-based CAT ligand immobilization also points to a specific interaction. In addition, kinetic analysis reveals strong binding, with the dissociation constant (K_D_) in the sub-micromolar range (8.6 × 10^–8^ M; Fig. 2c, Supplementary Table S2). This observation is further confirmed with isothermal titration calorimetry (ITC), revealing significant conformational changes occurring as a result of pAtPNP-A binding to bovine CAT (Supplementary Fig. S4).

### AtPNP-A interaction with CAT2 enhances catalase activity

**Figure 3:**
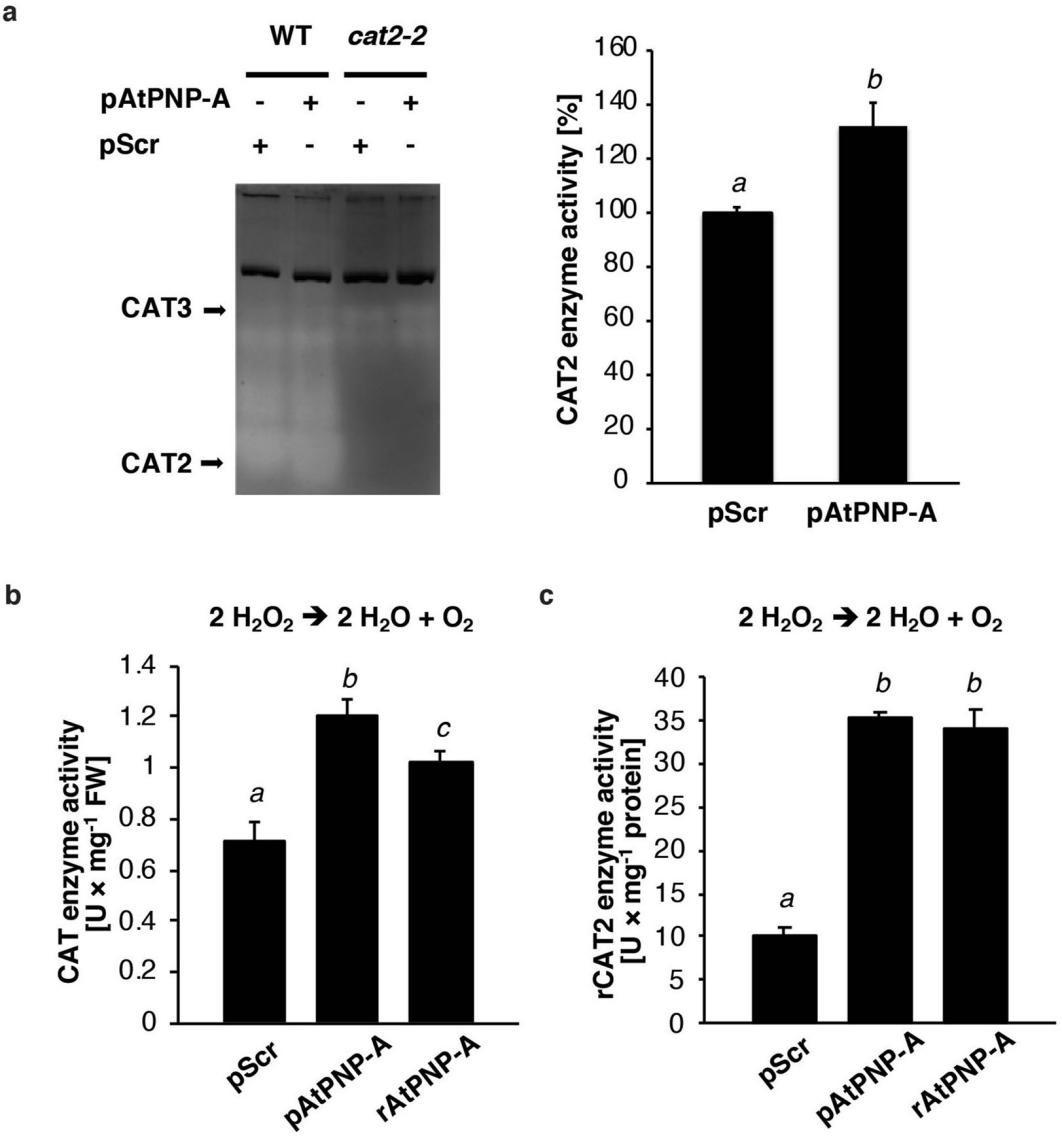
AtPNP-A directly interacts with CAT2 to modulate its enzymatic activity in vitro. (**a**) Zymogram depicting changes in the enzymatic activity of CAT isoforms extracted from wild type (WT) or *cat2-2* knockout mutant seedlings in response to 1 nM pAtPNP-A or pScr. Densitometric semi-quantification of bands corresponding to CAT2, normalized to the loading control (dark-coloured band on zymogram) for WT samples treated with 1 nM pAtPNP-A or pScr (mean ± SD, Student’s *t*-test, *n* = 3, *P* < 0.05). Different superscript (*a* and *b*) indicates significantly different results. (**b**) Total CAT activity in protein extracted from WT seedlings assayed with Amplex Red catalase assay kit in the presence of 1 nM pAtPNP-A, rAtPNP-A, or pScr. Different superscript (*a*, *b*, and *c*) indicates significantly different results (mean ± SD, one-way ANOVA, followed by Tukey–Kramer multiple comparison test, *n* = 3, *P* < 0.05). (**c**) Enzymatic activity of rCAT2 in the presence of 100 nM pAtPNP-A, rAtPNP-A, or pScr. Different superscript (*a* and *b*) indicates significantly different results from three independent experiments (mean ± SD, one-way ANOVA followed by Tukey–Kramer multiple comparison test, *n* = 3, *P* < 0.01).

To test whether the activity of CAT2 is modulated by AtPNP-A *in planta*, we investigated the basal levels of CAT activity, and the activity of CAT2 in particular, in response to AtPNP-A in WT seedlings and in seedlings with a mutated *CAT2* allele. To that end a homozygous mutant line carrying transfer DNA (T-DNA) insertions in *CAT2* is used. The *cat2-2* is a null mutant (T-DNA insertion located in the third exon; Supplementary Fig. S5a) that exhibits only residual (ca. 20%) extractable CAT enzymatic activity in leaves and less than 50% in roots^58–60^. The lack of CAT2 isoform in the *cat2-2* mutant plants was confirmed with native PAGE followed by specific CAT activity staining enabling determination of enzymatic activities of different CAT isoforms (Fig. 3a). Furthermore, exogenous application of nM concentrations of pAtPNP-A, in contrast to the treatment with pScr, results in increased CAT2 activity in WT plants compared to the mutants (Fig. 3a). This again confirms that CAT2 is an isozyme that is enzymatically modulated by AtPNP-A and is consistent with elevated total CAT activity of the protein extracts observed spectrophotometrically after incubation either with pAtPNP-A or the AtPNP-A recombinant (Fig. 3b). Since incubation of the total protein extracted from WT seedlings with pAtPNP-A and rAtPNP-A results in a more rapid H_2_O_2_ breakdown (Fig. 3b), we asked whether PNPs are capable of direct modulation of CAT activity. We noted that in vitro enzymatic activity of rCAT2 is significantly enhanced in the presence of rAtPNP-A and pAtPNP-A, but not pScr (Fig. 3c).

### AtPNP-A interacts with CAT2 in vivo

**Figure 4:**
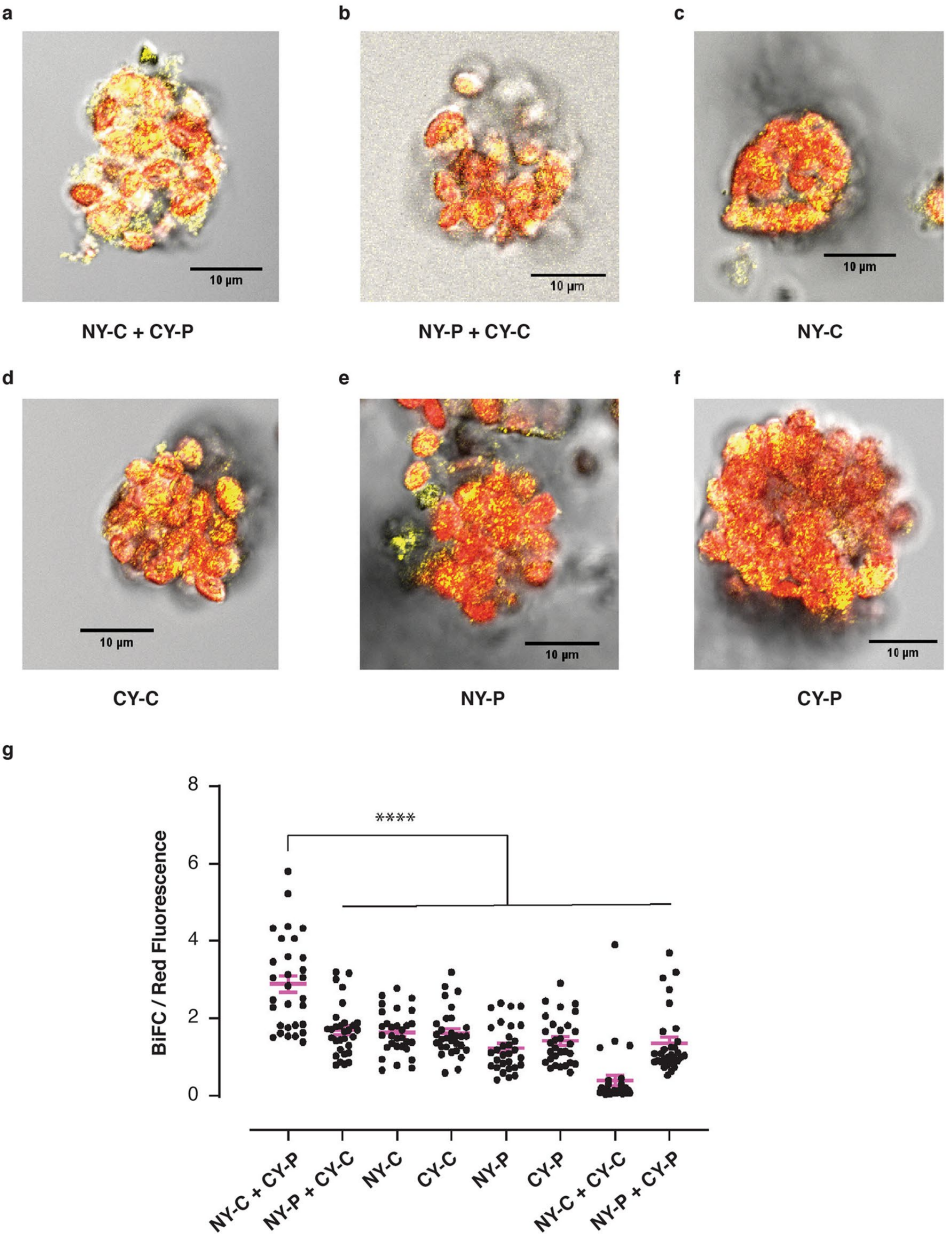
AtPNP-A interacts with CAT2 in vivo*.* (**a**)**–**(**f**) BiFC reveals AtPNP-A (indicated as P) and CAT2 (indicated as C) interact in the chloroplasts of tobacco leaf protoplasts. Exemplar merged images of protoplasts isolated from a leaf infiltrated with different combinations of N- or C-terminally tagged CAT2 and AtPNP-A are shown. (**g**) BiFC/Red fluorescence chloroplast analysis with mean ± SEM in pink. Normalized BiFC fluorescence is significantly higher in chloroplasts from protoplasts expressing NeYFP-CAT2, indicated as NY-C, and CeYFP-AtPNP-A, indicated as CY-P (mean ± SEM, one-way ANOVA followed by Sidak’s post-hoc test,* n* = 30, ****P < 0.0001).

Physical *in planta* association of AtPNP-A and CAT2 is confirmed with Bimolecular Fluorescence Complementation (BiFC) assay in tobacco leaf protoplasts (Fig. 4) revealing interaction of the proteins in chloroplasts of protoplasts (Fig. 4a, g). The greatest BiFC / Red fluorescence signal was quantified for interaction between NeYFP fused to CAT2 and CeYFP-tagged AtPNP-A (Fig. 4a) compared with other fusion configurations and controls (Fig. 4g). Lower BiFC/fluorescence signal observed for NeYFP-fused AtPNP-A and CeYFP-tagged CAT2 (Fig. 4b,g) interaction may reflect inhibition of the YFP reconstitution due to spatial restrictions of the three dimensional structure of the protein complex, or steric hindrance caused by the incorporation of the C/NeYFP, or misfolding. The organellar localization of the complementation is not surprising, considering the association of AtPNP-A with plastids and chloroplasts (Supplementary Fig. S1d,e). Notably, while CAT2 is predominantly a peroxisomal protein, its amino acid sequence includes predicted chloroplast transit peptide^61^ and increasing evidence suggests that it may participate in removal of H_2_O_2_ in subcellular compartments other than peroxisomes^40,62,63^.

**Figure 5:**
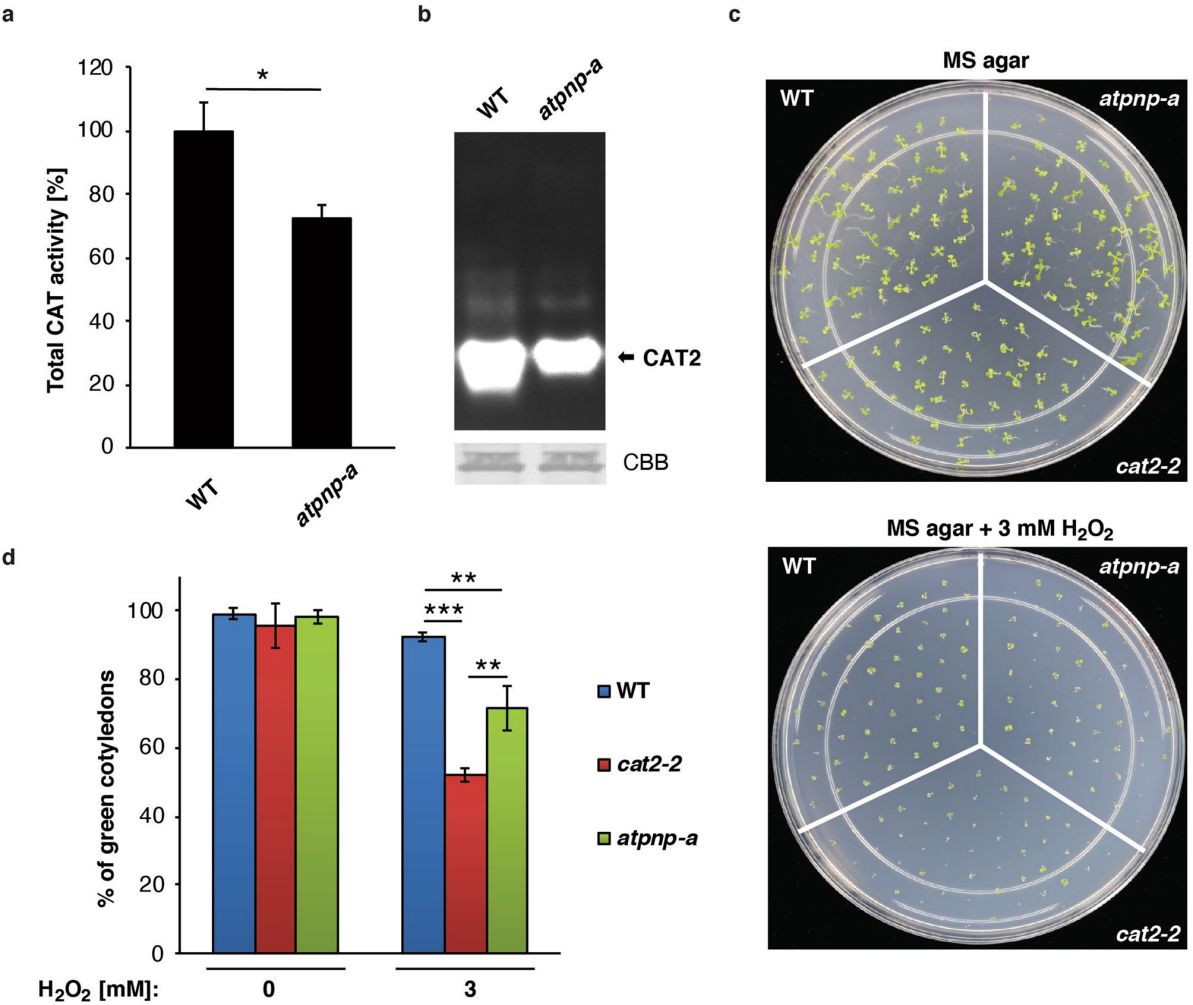
The *atpnp-a* mutant plants phenocopy *CAT2*-deficient plants in their ability to cope with H_2_O_2_ stress. (**a**) Total CAT enzymatic activities in equal amounts of protein extracted from leaves of 4 week-old WT and *atpnp-a* mutant seedlings. The graph shows data from three independent experiments (mean ± SD, Student’s *t*-test, *n* = 3, * *P* = 0.0409). (**b**) Zymogram of CAT isoform activities in protein extracts from *atpnp-a* or WT seedlings separated in 8% native PAGE and stained specifically for CAT activity. Coomassie brilliant blue (CBB) shows equal loading. Full-length gels are presented in Supplementary Fig. S5a. (**c**) Germination of WT, *cat2-2*, and *atpnp-a* seeds 14 days after sowing on MS agar supplemented with 3 mM H_2_O_2_. (**d**) Quantification of germination (as shown in section (**c**)) by the presence of green cotyledons. The graph shows data from three independent experiments (mean ± SD, one-way ANOVA followed by Tukey–Kramer multiple comparison test, *n* = 300, ** *P* < 0.01, *** *P* < 0.001).

Next, we asked a question whether CAT2 interaction with AtPNP-A is of physiological significance. To test whether CAT2 activity is affected by AtPNP-A *in planta*, we examined basal levels of CAT activity, and the activity of CAT2 in particular, using seedlings of a homozygous mutant line carrying T-DNA insertion in *AtPNP-A*. The *atpnp-a* mutant is a knockdown line with a T-DNA insertion located in the second intron of *AtPNP-A* (Supplementary Fig. S5b). Extractable CAT activity is approximately 20% lower in leaves of *atpnp-a* than in WT plants (Fig. 5a) and the mutant seedlings display decreased activity of the CAT2 isoform (Fig. 5b). The interaction of CAT2 and AtPNP-A is physiologically relevant in germination assays, where *atpnp-a* phenocopy *cat2-2* mutant seeds in impaired growth in the presence of H_2_O_2_ (Fig. 5c-d). The impaired germination and growth of seedlings due to their sensitivity to H_2_O_2_ is indicative of a disturbance in cellular redox homeostasis in these mutant lines.

## Discussion

PNPs are implicated in plant responses to biotic^32,64^ and abiotic stresses^13,65^, and partially may do so by modulating cellular levels of ROS^20^. Pre-treatment of leaf discs with PNP prior to treatment with flg22 enhances oxidative burst (Supplementary Fig. S2), indicating that PNPs temper host resistance against pathogens such as *Pst* DC3000^16,17^ at least in part, by modulating redox signaling. Whereas it is plausible that PNPs affect redox signaling in multiple ways in different cell types under specific conditions, here we reveal that one of these mechanisms involves a direct interaction of AtPNP-A with CAT2 (Table 1, Fig. 2a,b). In vivo interaction between these proteins is observed by co-localization in chloroplasts (Fig. 4), pointing at compartmentalization of cellular processes modulated by AtPNP-A that involve regulation of ROS homeostasis, and is consistent with cellular localization of PNPs (Supplementary Fig. S1d,e). Binding of AtPNP-A to CAT2 enhances its enzymatic activity (Fig. 3). The *atpnp-a* knockdown phenocopies *CAT2*-deficient mutant seeds with compromised sensitivity to H_2_O_2_ (Fig. 5c-d). This may result from decreased extractable total CAT activity (Fig. 5a), and CAT2 activity in particular (Fig. 5b), in the *atpnp-a* knockdown line compared to the WT plants. The specific AtPNP-A and CAT2 interaction in vitro (Fig. 2a, Supplementary Fig. S3b,c) and in vivo (Fig. 4) lend support to the idea that the differences in CAT2 activity result from direct interaction of the enzyme with AtPNP-A. Taken together, these results indicate that AtPNP-A may affect plant responses to abiotic stresses, including response to oxidative stress during germination (Fig. 5c,d), by modulating redox homeostasis via direct interaction with CAT2.

Several lines of evidence indicate AtPNP-A has a protective role in the response to infection with *P. syringae*^16,17^. Our results suggest that this may be a consequence of an augmented oxidative burst generated upon perception of elicitors, such as flg22, in the presence of PNPs (Supplementary Fig. S2), since exogenous H_2_O_2_ enhances plant pathogen resistance^66^. This is also consistent with an AtPNP-A function in hyper-activation of signaling mediated by plasma membrane-localized pattern recognition receptors (PRRs) that perceive pathogen-derived molecules^4^, and with its perception by receptors^16,21^. Like animal PRRs, plant PRRs activate innate immune responses to fend off pathogens^4^. The enhanced flg22-induced oxidative burst observed upon pre-incubation of leaf discs with AtPNP-A not only supports the protective function of PNPs against pathogens by enhancing plant innate immunity^17^, but also indicates that it does so by modulating redox signaling. Nevertheless, the process by which AtPNP-A pre-treatment of leaf discs enhances flg22-oxidative burst is far from resolved. In contrast to flg22, pAtPNP-A alone does not activate mitogen-activated protein kinase (MAPK) pathway (data not shown), but PNP could for instance affect accumulation of some of its components, either at the transcript or protein level^20^, priming the plant for subsequent pathogen attack and thereby enabling a quicker response by generating a more pronounced radical burst e.g. via the modulation of the plasma membrane NADPH oxidase by AtPNP-A. However, neither NADPH oxidases nor peroxidases, enzymes responsible for ROS generation upon pathogen attack^67^, have been identified in our protein–protein interaction studies, although animal analogues of PNP have been shown or speculated to activate NADPH oxidase^68,69^. Instead, we identified CAT2 (Fig. 1c, Table 1), an enzyme crucial for scavenging excess H_2_O_2_ produced during pathogenesis^70^, as a binding partner of AtPNP-A.

Importantly, plant pathogens can activate CATs in response to oxidative bursts, either to strengthen pathogen cell walls or to compromise ROS-mediated host defense, and in many cases CATs have been identified as virulence factors, e.g. in *P. syringae* DC3000^71^. In *Xanthomonas axonopodis* pv. *citri*, a PNP-like protein (XacPNP) is induced in the pathogen during the plant-based stages of the life cycle^64^. PNP-like molecules are expressed by several plant pathogens^64,72^ and are considered a double-edged sword for both partners in the plant-pathogen arms race.

A PNP—CAT interaction in plant responses to infecting pathogens is not surprising. CATs commonly function to down-regulate ROS during ABA-induced stomatal closure, and ABA-induced stomatal closure can be inhibited by removal of H_2_O_2_ via CAT2^73,74^. AtPNP-A has been shown to significantly delay and reduce the extent of ABA-induced stomatal closure, while ABA had no effect on either AtPNP-A-dependent guard cell volume increases or AtPNP-A-dependent cGMP increases^13^. Therefore, it is tempting to speculate that AtPNP-A modulates the effect of ABA on stomatal aperture through the interaction with CAT2 (and/or other CATs), enhancing its enzymatic activity in vivo as part of the *in planta* function.

It is also conceivable that modulation of CAT2 activity by AtPNP-A may not only have implications for plant biotic stress tolerance but may also result in changes in responses to abiotic stress. Activity of CAT2 is required for decomposition of photorespiration-derived H_2_O_2_ preventing redox perturbation under ambient growth conditions^59^ while CAT2 deficiency causes a defective photorespiration phenotype including suppressed growth^58,59^ and accumulation of H_2_O_2_ in leaves^75^. During senescence suppression of *CAT2* function, mediated by G-BOX BINDING FACTOR1 (GBF1)^76^, decreases CAT2 activity and increases ROS accumulation^77^. Interestingly, *AtPNP-A* is a senescence-enhanced gene^65^ and *atpnp-a* knock-down seedlings display premature senescence phenotype (data not shown), thereby phenocopying *cat2* mutant plants^59^. Increases in *CAT2* transcript were observed upon dehydration^78^ as well as exogenous application of SA, H_2_O_2_, and a superoxide-generating herbicide methyl viologen^56^, with the latter causing extensive chlorosis in *AtPNP-A*-deficient seedlings^17^.

On a more general level, it is not surprising that many hormone receptors have enzymatic functions, since ligand binding needs to directly translate into a molecular response. Activation of kinase receptors by ligand binding is followed by consequent specific phosphorylation events. Natriuretic peptide receptors A and B contain cytosolic guanylate cyclase domains activated upon ligand binding^79^. This ligand-dependent activation of the cyclase causes the generation of cGMP, which in turn acts as second messenger for the multiple downstream responses. Given the perhaps surprising similarities between the vertebrate and the plant NP receptors, our result begs the question if possibly vertebrate NPs can also modulate vertebrate catalases. Realization that AtPNP-A is capable of undergoing specific in vitro interaction with monofunctional CAT proteins derived from different sources, including bovine (Fig. 2c, Supplementary Fig. S4), prompts speculation that not only CAT2 interacts with PNPs, but it is possible that the interaction between NPs and CAT proteins is not exclusive to the plant kingdom and may be prevalent in other systems.

In summary, we present evidence for a specific and direct interaction between AtPNP-A and CAT2 and propose that the AtPNP-A modulated CAT2 activity ensures the maintenance of an optimized redox state during plant stress responses. Since CAT2 is not limited to protecting cells from H_2_O_2_, but also modulates its level to maintain optimal redox states of antioxidants, including ascorbate or glutathione, our study on PNP-dependent modulation of CAT2 activity may inform further research into H_2_O_2_ signaling and whole cell reduction–oxidation homeostasis.

## Supplementary information

Supplementary Informations.

## Data Availability

Complete proteomics data set has been published in^44^.
