# Supplementary material for: A natriuretic peptide from Arabidopsis thaliana (AtPNP-A) can modulate catalase 2 activity
Source: Sci Rep. 2020 Nov 12;10:19632. doi: 10.1038/s41598-020-76676-0 (PMC7665192; doi:10.1038/s41598-020-76676-0)
Supplement: Supplementary file 1 — Supplementary Informations. [file 41598_2020_76676_MOESM1_ESM.pdf]

Supplementary Information for

A natriuretic peptide from *Arabidopsis thaliana* (AtPNP-A) can modulate catalase 2 activity

Turek I, Wheeler J, Bartels S, Szczurek J, Wang JH, Taylor P, Gehring C, Irving H

The following pages include:

- Supplementary Tables 1 and 2
- Supplementary Figures 1 to 6
- Reference list

Supplementary Table S1. Sequences of PCR primers used for genotyping of mutant alleles and isolation of gene constructs.

Primers for genotyping.

Allele	Name	Sequence (5′- 3′)
WT At2g18660	LP	TTTCCGGATATCCGAAATTTC
WT At2g18660	RP	ATGTGATTTAGGGTCTGCGTG
Mutant At2g18660	LBb1.3	ATTTTGCCGATTTCGGAAC
Mutant At2g18660	RP	ATGTGATTTAGGGTCTGCGTG
WT At4g35090	LP	ACATTTTGGAGCATTGACTGG
WT At4g35090	RP	TCTGGTGCTCCTGTATGGAAC
Mutant At4g35090	LBb1.3	ATTTTGCCGATTTCGGAAC
Mutant At4g35090	RP	TCTGGTGCTCCTGTATGGAAC

Wild type (WT) alleles were determined by PCR with a pair of genomic left primer (LP) and right primer (RP) corresponding to the gene of interest. T-DNA insertion mutant alleles were determined by PCR with a pair of locus specific RP and a T-DNA left boarder (LB) primer (LBb1.3). The primers were designed with the T-DNA primer design tool (http://signal.salk.edu/tdnaprimers.2.html) to identify T-DNA insertion mutant plants homozygous for *AtPNP-A* (At2g18660) or mutant plants homozygous for *CAT2* (At4g35090). Determination of the site of the T-DNA insertion in the *atpnp-a* homozygous mutant line was done by sequencing of the purified PCR product obtained with the use of the mutant allele-specific primers.

Supplementary Table S2. Kinetics parameters of the interaction between pAtPNP-A and bovine liver CAT.

a. Kinetics report

k_a [1/Ms] (±SE)	1.089 x 10^4 (±68)
k_d [1/s] (±SE)	9.415 x 10^{-4} (±3.7 x 10^{-6})
K_D [M]	8.646 x 10^{-8}
R_{max} [RU] (±SE)	125 (±0.13)
Flow [μL/min]	100
Chi^2 [RU^2]	10.4
U-value	2
tc (±SE)	1.169 x 10^7 (±3.7 x 10^6)
k_t [RU/Ms]	5.427 x 10^7

b. Parameters of the binding cycles

Concentration [M]	RI [RU]	SE (RI)
7.390 × 10^{-9}	3.242	0.056
1.478 × 10^{-8}	4.510	0.074
2.956 × 10^{-8}	7.073	0.099
5.912 × 10^{-8}	11.06	0.11
1.182 × 10^{-7}	16.52	0.12
2.365 × 10^{-7}	24.75	0.13
4.730 × 10^{-7}	36.05	0.13
9.459 × 10^{-7}	46.29	0.14
1.892 × 10^{-6}	70.57	0.14
3.784 × 10^{-6}	96.47	0.14
7.390 × 10^{-9}	3.379	0.049

Abbreviations: k_a – association rate constant, k_d – dissociation rate constant, K_D – equilibrium dissociation constant, R_{max} – maximum response units reached at equilibrium, k_t – mass transfer constant, tc – flow-rate independent component of the mass transfer constant, Chi^2 – average squared residual, U-value – uniqueness value for kinetic rate constants, SE – standard error, RI – refractive index, RU – resonance unit.

a

```
AtPNP-A     --PYTRSACYGTQRE-TLVVGVKNNLWQNGRACGRRY--
NtPNP-A     --QYVPSACYGNTPQGTIIAAASDPLWNNGAICGKTF--
StPNP-A     --NYVPSACYGSTPQGTIIAAASDPLWADGKICGKTF--
                *. *****.   : *::....: ** :*  **: :
```

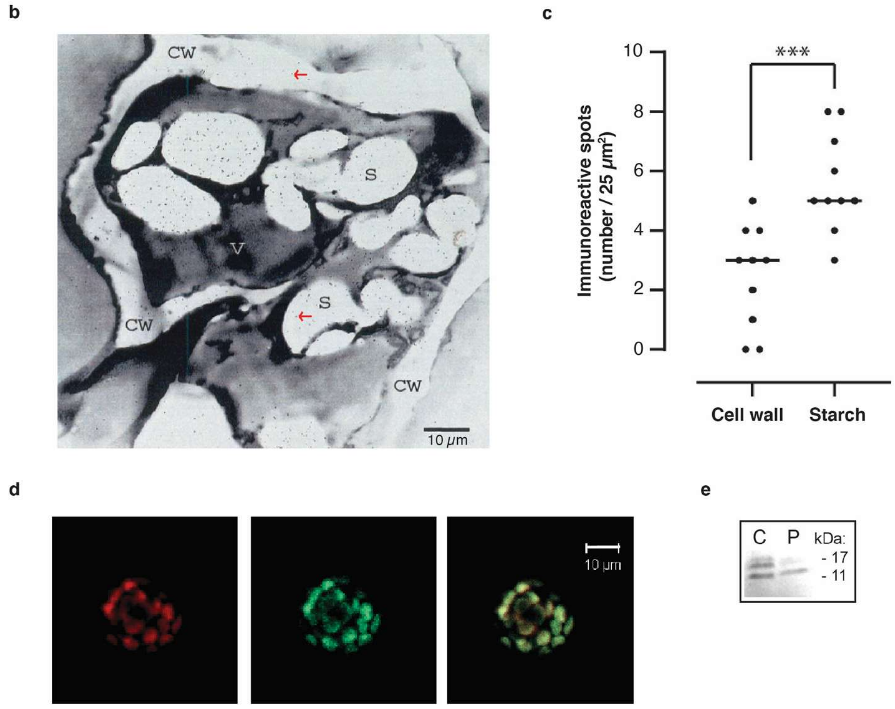

Supplementary Figure S1. Extra- and intracellular localization of PNP.

(a) Amino acid sequence alignment of the active site of *A. thaliana* PNP (AtPNP-A), *N. tabaccum* (NtPNP-A), and *S. tuberosum* PNP (StPNP-A). Identical residues are indicated with an asterisk (*), conserved residues with a colon (:), and semi-conserved residues with a full stop (.). Alignments of PNP-like proteins from other species can be found in[1-6]. (**b**) Electron micrograph of potato showing gold-labeled anti-hANP immunodots in the cell wall (CW) and starch bodies (S). V – vacuole. Red arrows indicate exemplar immunodots. Scale bar = 10 µm. (**c**) Quantitative analysis of the immunoreactive spots detected in cell wall or starch bodies. Immunoreactive dots in 10 5x5 µm squares in cell wall and starch bodies were counted and analyzed (median ± SEM, unpaired Student's *t*-test, $n = 3$, *** $P < 0.0006$). (**d**) Confocal microscopy image showing colocalization of GFP:signalAtPNP-A fusion protein (green) and chloroplasts (red) 18 h after transfection. Scale bar = 10 µm. (**e**) Immunodetection of AtPNP-A in protoplasts (P) and chloroplasts (C) isolated from wild type Arabidopsis leaves with an antibody raised to a unique region in AtPNP-A (amino acids 44-55)[7]. Immunoblot is presented in Supplementary Fig. S6b.

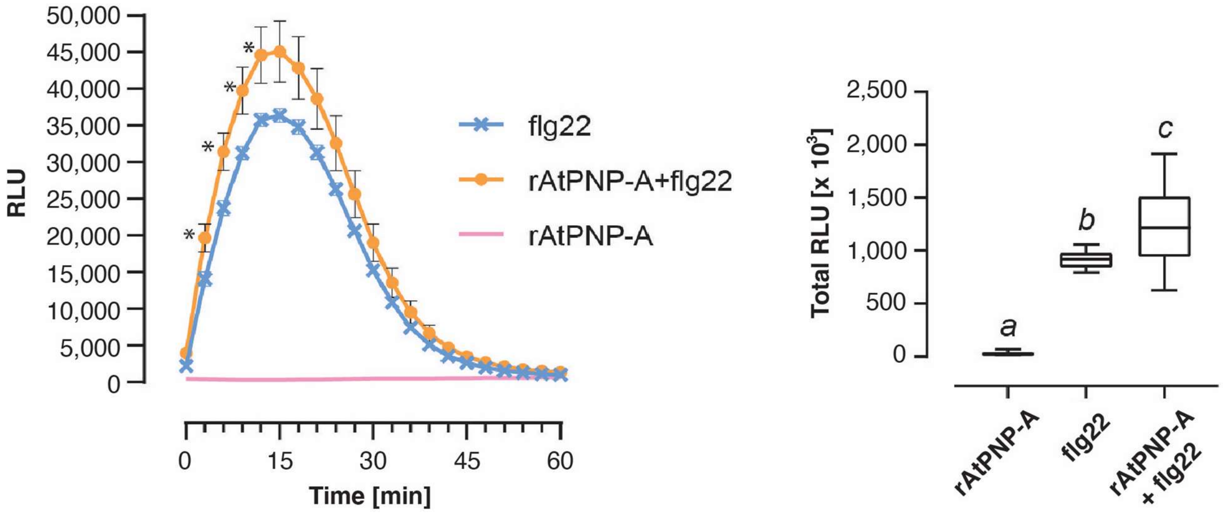

Supplementary Figure S2. AtPNP-A participates in plant immunity.

Kinetics of ROS production after treatment of leaf discs with 1 µM flg22 with or without pre-treatment with 100 nM recombinant AtPNP-A (rAtPNP-A), or after treatment with rAtPNP-A only. Data are shown as mean ± SEM (two-way repeated measure ANOVA followed by Tukey's multiple comparison test, $n = 11 – 12$, * $P < 0.01$). Total production of ROS during 60 min after treatment with rAtPNP-A, flg22, or flg22 and rAtPNP-A pre-treatment is shown as median ± SD (one-way ANOVA and Tukey-Kramer multiple comparison test, $n = 11 – 12$, $P < 0.01$) with statistically significant differences indicated by different letters.

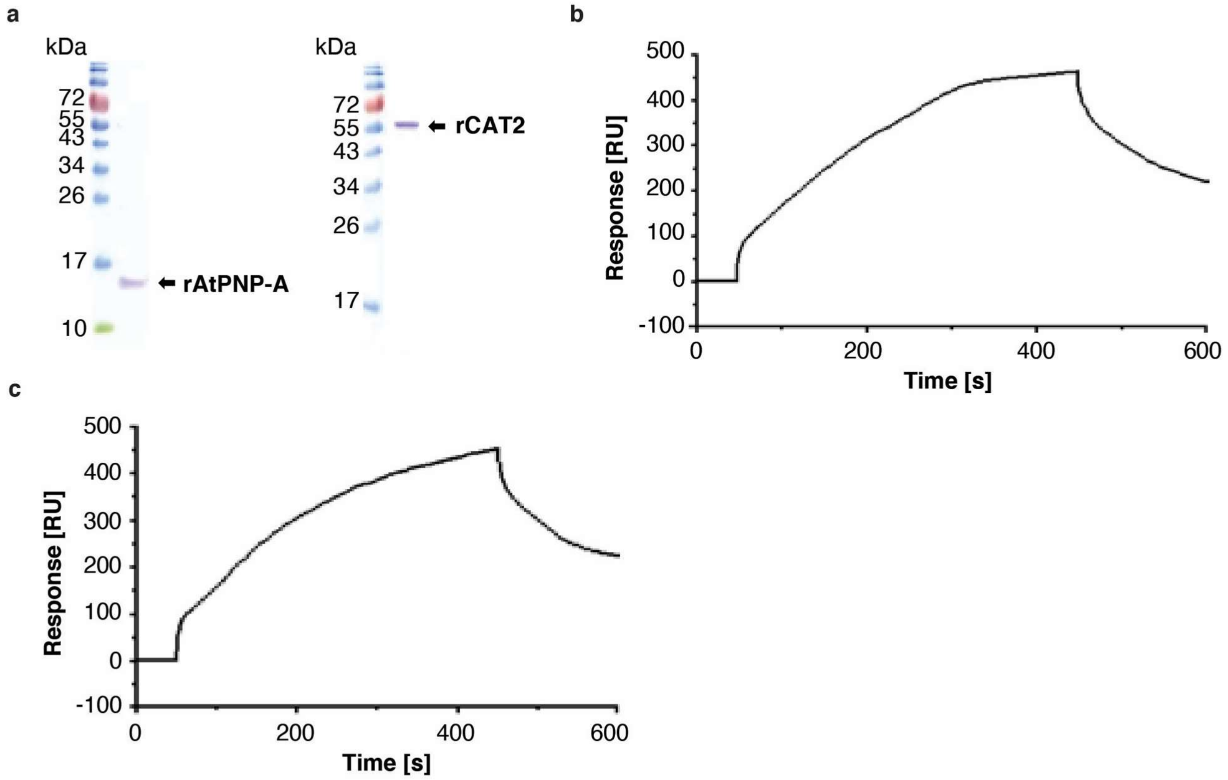

Supplementary Figure S3. *In vitro* association of AtPNP-A and CAT2 recombinant proteins. (**a**) Recombinant protein preparations used in SPR analyses. (**b**) – (**c**) Exemplar sensorgrams depicting referenced binding response of the CAT2 and AtPNP-A recombinant proteins. Purified rCAT2 was immobilized using amine-coupling chemistry on the active surface of the CM5 sensor chip, while the reference surface was blank activated and did not carry any ligand, and the purified rAtPNP-A was used as an analyte (**b**). The binding was verified in inverted configuration using the CM5 sensor chip, with the purified rAtPNP-A immobilized using amine-coupling chemistry on the active surface and the purified rCAT2 used as an analyte (**c**).

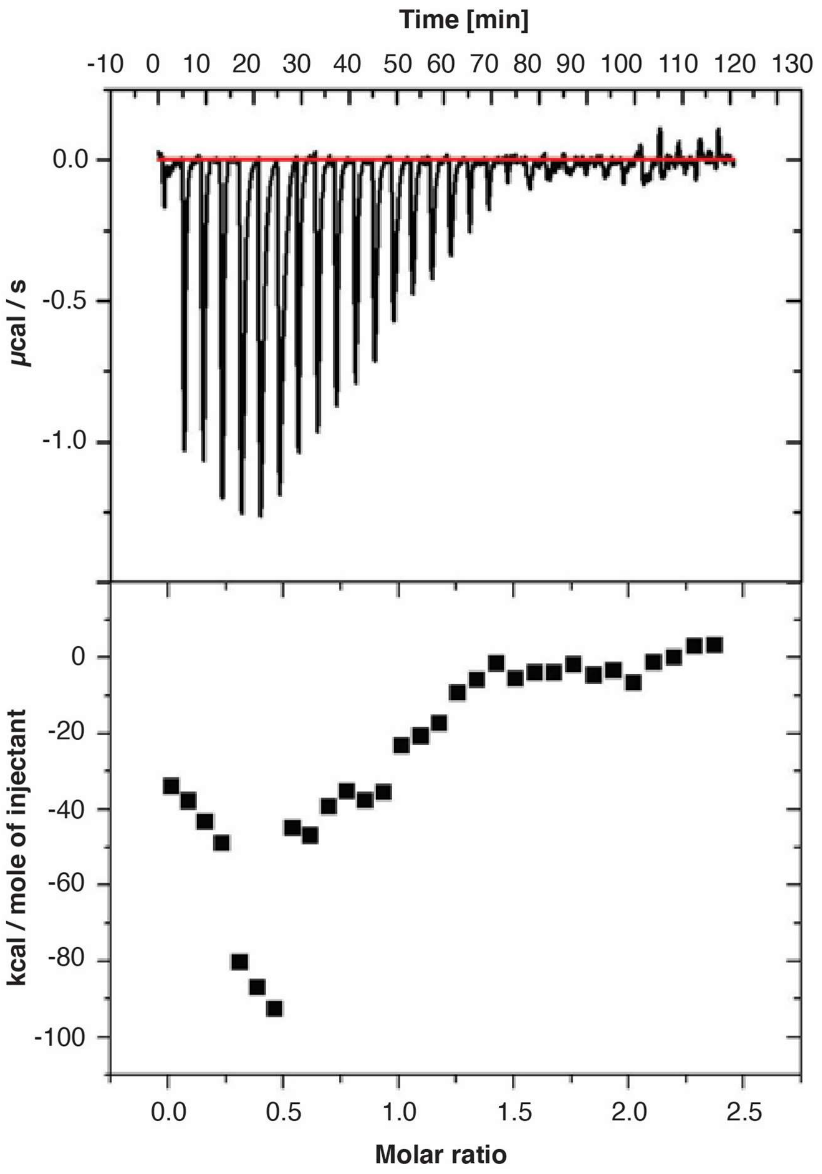

Supplementary Figure S4. *In vitro* association of pAtPNP-A and bovine liver CAT assessed with isothermal titration calorimetry (ITC).
Raw (upper panel) and integrated (bottom panel) data revealing significant conformational change occurring during the binding of pAtPNP-A to CAT from bovine liver.

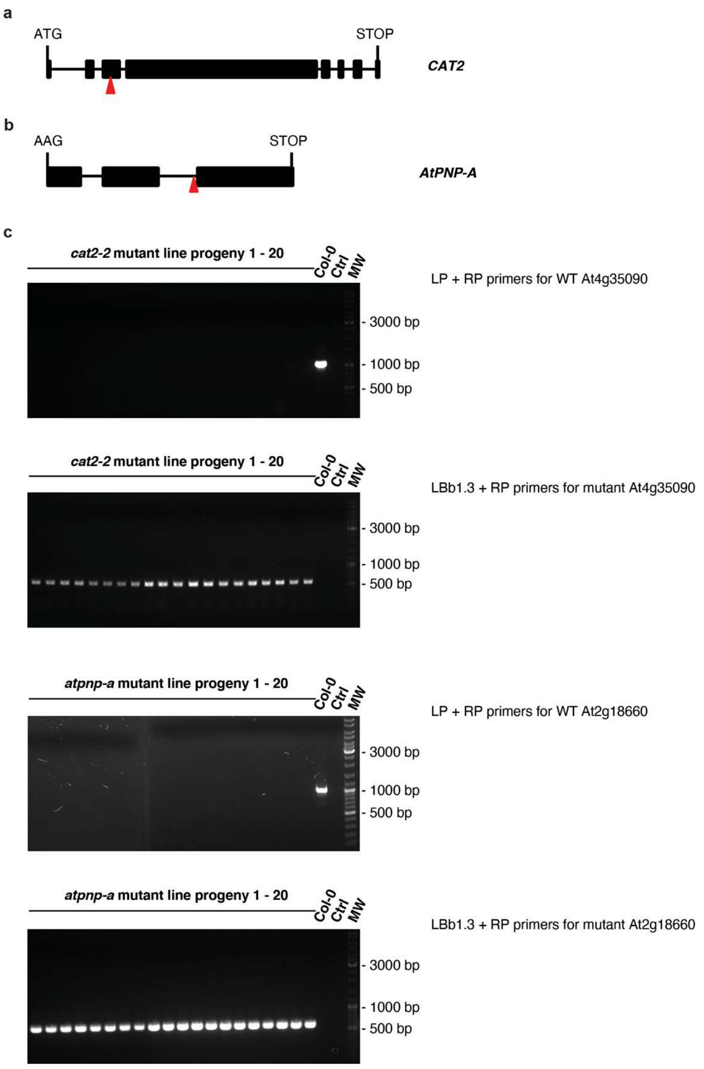

Supplementary Figure S5. Position of the T-DNA insertion sites within genome of *atpnp-a* and *cat2-2* mutants.

(**a**) Genomic organization of the *CAT2* gene and the localization of the T-DNA insertion sites within *cat2-2*, as determined by PCR and sequencing of the flanking regions. (**b**) Genomic organization of the *AtPNP-A* gene and the localization of the T-DNA insertion sites within *atpnp-a*, as determined by PCR and sequencing of the flanking regions. Black boxes represent exons, bold lines correspond to introns and the insertions are indicated with arrows. (**c**) Images of agarose gels showing results of PCR screening undertaken to identify homozygous WT, homozygous mutant and heterozygous individual plants from the segregating seed stocks[8] using primers (LP, RP, LBb1.3) listed in Supplementary Table S1. Ctrl – 'no template' negative control, MW – molecular weight DNA ladder.

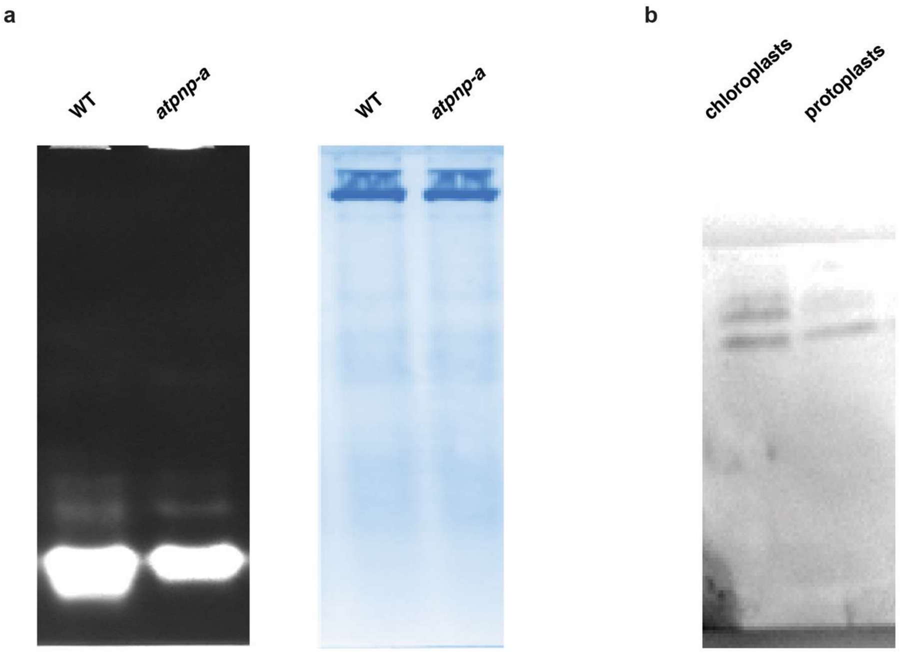

Supplementary Figure S6. Images of native gels and immunoblot.
(**a**) Full-length native PAGE gels corresponding to the cropped zymogram and Coomassie brilliant blue-stained native gel shown in Fig. 5b. (**b**) Immunoblot corresponding to the cropped immunoblot shown in Supplementary Fig. S1e.

Reference List for Supplementary Information

1. Ludidi, N. N., Heazlewood, J. L., Seoighe, C., Irving, H. R. & Gehring, C. A. Expansin-like molecules: novel functions derived from common domains. *J. Mol. Evol.* **54**, 587–594 (2002).
2. Rafudeen, S. *et al.* A role for plant natriuretic peptide immuno-analogues in NaCl- and drought-stress responses. *Physiol. Plant.* **119**, 554–562 (2003).
3. Maryani, M. M. *et al. In situ* localization associates biologically active plant natriuretic peptide immuno-analogues with conductive tissue and stomata. *J. Exp. Bot.* **54**, 1553–1564 (2003).
4. Nembaware, V., Seoighe, C., Sayed, M. & Gehring, C. *Xanthomonas axonopodis* may induce hyper-hydration in the plant host: a hypothesis of molecular mimicry. *BMC Evol. Biol.* **4**, 10 (2004).
5. Morse, M., Pironcheva, G. & Gehring, C. AtPNP-A is a systemically mobile natriuretic peptide immunoanalogue with a role in *Arabidopsis thaliana* cell volume regulation. *FEBS Lett.* **556**, 99–103 (2004).
6. Gottig, N. *et al. Xanthomonas axonopodis* pv. *citri* uses a plant natriuretic peptide-like protein to modify host homeostasis. *Proc. Natl. Acad. Sci. USA* **105**, 18631–18636 (2008).
7. Wang, Y. H., Ahmar, H. & Irving, H. R. Induction of apoptosis by plant natriuretic peptides in rat cardiomyoblasts. *Peptides* **31**, 1213–1218 (2010).
8. Østergaard, L. & Yanofsky, M. F. Establishing gene function by mutagenesis in *Arabidopsis thaliana*. *Plant J. Cell Mol. Biol.* **39**, 682–696 (2004).
